# Unique roles of *Akt1* and *Akt2* in IGF-IR mediated lung tumorigenesis

**DOI:** 10.18632/oncotarget.6489

**Published:** 2015-12-07

**Authors:** S. Elizabeth Franks, Ritesh Briah, Robert A. Jones, Roger A. Moorehead

**Affiliations:** ^1^ Department of Biomedical Science, Ontario Veterinary College, University of Guelph, Guelph, Ontario, Canada

**Keywords:** Akt, lung cancer, IGF-IR, transgenic mice, tumor development

## Abstract

AKT is a serine-threonine kinase that becomes hyperactivated in a number of cancers including lung cancer. Based on AKT's association with malignancy, molecules targeting AKT have entered clinical trials for solid tumors including lung cancer. However, the AKT inhibitors being evaluated in clinical trials indiscriminately inhibit all three AKT isoforms (AKT1–3) and it remains unclear whether AKT isoforms have overlapping or divergent functions. Using a transgenic mouse model where IGF-IR overexpression drives lung tumorigenesis, we found that loss of *Akt1* inhibited while loss of *Akt2* enhanced lung tumor development. Lung tumors that developed in the absence of *Akt2* were less likely to appear as discrete nodules and more frequently displayed a dispersed growth pattern. RNA sequencing revealed a number of genes differentially expressed in lung tumors lacking *Akt2* and five of these genes, *Actc1*, *Bpifa1, Mmp2, Ntrk2*, and *Scgb3a2* have been implicated in human lung cancer. Using 2 human lung cancer cell lines, we observed that a selective AKT1 inhibitor, A-674563, was a more potent regulator of cell survival than the pan-AKT inhibitor, MK-2206. This study suggests that compounds selectively targeting AKT1 may prove more effective than compounds that inhibit all three AKT isoforms at least in the treatment of lung adenocarcinoma.

## INTRODUCTION

Lung cancer is the leading cause of cancer-related mortalities worldwide [1, 2]. Lung cancer is often not detected until advanced stages of the disease [3] where the 5 year survival rate is close to 1% [4] despite advances in treatment and the incorporation of new drugs and targeted therapies. Therefore, the development of new therapeutics for the treatment of lung cancer is critical for patients facing this illness. AKT, also known as protein kinase B (PKB), regulates a diverse set of cellular functions relevant in the growth and progression of lung cancer cells, including proliferation [5], survival [6, 7], migration and invasion [8–10]. Furthermore, AKT is an important mediator of the PI3K/AKT/mTOR pathway, which is a common signaling pathway used by several growth factors and cytokines that can be involved in promoting cancer growth and progression [11, 12]. This makes targeting the PI3K/AKT/mTOR pathway an appealing therapeutic strategy in order to combat the clinical challenges of tumor heterogeneity and acquired resistance [13]. Indeed, AKT targeted drugs are currently in development to treat several types of cancer, including lung cancer [14, 15].

Increased activation of AKT is frequently observed in small cell lung cancer (SCLC) [16] and across all subtypes of non-small cell lung cancer (NSCLC) [17–19]. High levels of activated AKT in NSCLC is associated with decreased survival [18, 20–23] and has been specifically associated with higher grade and stage [24, 25] as well as lymph node metastasis [26]. AKT is also frequently detected in early stage NSCLC [27] and metaplastic/dysplastic pre-lesions [8, 28–30] and may be associated with tumorigenesis from exposure to air pollution [30] and tobacco carcinogens [31–33].

AKT exists as three isoforms (AKT1–3 or PKBα/β/γ), which are encoded by separate genes located on different chromosomes [11, 34]. However, the proteins have a high sequence and structural similarity and contain an N-terminal PH domain followed by a linker region, a kinase domain, and a short C-terminal hydrophobic motif [34, 35]. While it was previously thought that the three AKT isoforms were redundant in activity, evidence has emerged supporting isoform specific roles. Isoform specific knockout mice demonstrate that *Akt1* is primarily involved in somatic growth [36], *Akt2* is the prominent isoform involved in glucose metabolism [37, 38], and *Akt3* is only critical in neuronal development [39]. Unique roles for AKT isoforms in tumorigenesis have been observed in mouse models of breast [40] and lung [41, 42] cancers. There are several studies indicating isoform differences in lung cancer cell migration [43, 44], proliferation [45], and epithelial-mesenchymal transition [46]. Furthermore, high levels of intra-tumoral AKT2 in NSCLC patients was associated with increased survival but activated AKT was a negative prognosticator of survival [47], indicating AKT2 may have an opposing, protective role. Despite growing evidence of differing roles, there are inconsistencies in the literature and the roles of AKT isoforms within lung cancer remain poorly understood.

Our lab has generated a doxycycline-inducible, tissue-specific transgenic mouse model of lung cancer [48]. Using a surfactant protein C (SPC) promoter, the type I insulin-like growth factor receptor (IGF-IR) is overexpressed in type II alveolar cells which initiates the development of tumors in the lungs. These lung tumors typically present as one or more discrete nodules on the surface of the lung and express high levels of activated AKT [48]. To investigate the role of specific AKT isoforms in lung tumorigenesis, these SPC-IGFIR transgenic mice were crossed with *Akt1* null or *Akt2* null mice to generate SPC-IGFIR-*Akt1^−/−^* and SPC-IGFIR-*Akt2^−/−^*

## RESULTS

### Lung tumorigenesis is augmented in SPC-IGFIR-*Akt2^−/−^* mice and suppressed in SPC-IGFIR-*Akt1^−/−^* mice

**Figure 1:**
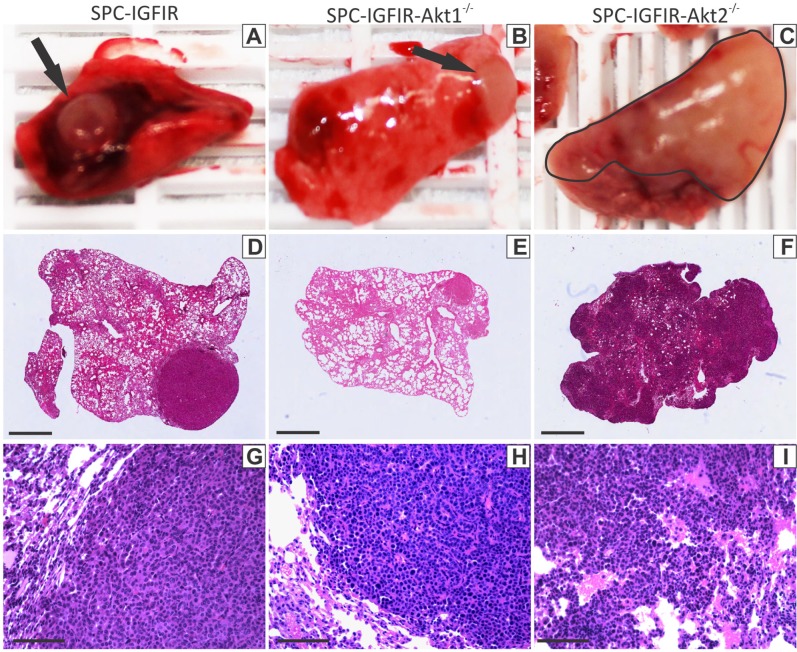
Loss of *Akt2* alter lung tumor appearance Representative macroscopic images of lung tumors that developed in SPC-IGFIR mice **A.** SPC-IGFIR-*Akt1^−/−^* mice **B.** and SPC-IGFIR-*Akt2^−/−^* mice **C.** Distinct nodular tumors are visible in lungs from SPC-IGFIR (A, arrow) and SPC-IGFIR-*Akt1^−/−^* (B, arrow) mice while tumors display a more dispersed distribution pattern in SPC-IGFIR-*Akt2^−/−^* mice (C, black outline). This pattern of nodular or disperse tumor growth is also apparent on histological sections of lungs from SPC-IGFIR mice **D, G.** SPC-IGFIR-*Akt1^−/−^* mice **E, H.** and SPC-IGFIR-*Akt2^−/−^* mice **F, I.** Error bars in D-F, 0.5 mm and G-I 100 μm.

Based on tumor kinetics of our previous studies [48, 49] we had anticipated treating all mice for 9 months with rodent chow containing 2g of doxycycline/kg chow (to induce IGF-IR transgene expression). However, SPC-IGFIR-*Akt2^−/−^* mice displayed characteristics associated with poor health (i.e. ruffled coat, labored breathing, hunched posture, etc) after 8 months of IGF-IR overexpression. Necropsy of the SPC-IGFIR-*Akt2^−/−^* mice revealed extensive tumor development following 8 months of IGF-IR overexpression (Figure 1C, 1F) and thus SPC-IGFIR-*Akt2^−/−^* mice were collected at this time point. A subset of SPC-IGFIR mice were also collected at this time point to serve as controls. The remaining SPC-IGFIR and SPC-IGFIR-*Akt1^−/−^* mice were collected after 9 months of IGF-IR overexpression.

Overexpression of IGF-IR was sufficient to cause lung tumor development in all mice (Table 1). These lung tumors were visible macroscopically on the lung surface as white, round nodules in SPC-IGFIR (Figure 1A, arrow) and SPC-IGFIR-*Akt1^−/−^* mice (Figure 1B, arrow), however, the white solid tumor tissue in SPC-IGFIR-*Akt2^−/−^* mice (Figure 1C, black outline) was less likely to retain the nodular shape and appeared to have more extensive growth into the lung. The number of visible surface tumors in SPC-IGFIR and SPC-IGFIR-*Akt1*^−/−^ mice were counted and SPC-IGFIR-*Akt1*^−/−^ mice had significantly fewer surface tumors than SPC-IGFIR mice (Table 1). The lack of discrete nodules prevented the counting of surface tumors in SPC-IGFIR-*Akt2*^−/−^ mice. Microscopically, similar phenotypes were observed in H&E stained tissues (Figure 1D–1I). While some small tumors in SPC-IGFIR-*Akt2^−/−^* mice appear nodular, tumors frequently lacked defined boundaries (Figure 1I) of the nodular tumors observed in the SPC-IGFIR (Figure 1G) and SPC-IGFIR-*Akt1^−/−^* (Figure 1H) mice.

**Table 1:** Tumor Characteristics

Genotype	Tumor Incidence	Number of Visible Surface Tumors	Tumor Area (% of lung area)
SPC-IGFIR (9 months)	100%	14.6 ± 1.9	17.8 ± 2.9
SPC-IGFIR-Akt1^−/−^ (9 months)	100%	5.6 ± 1.2^a^	7.1 ± 1.3^a^
SPC-IGFIR (8 months)	100%	11.1 ± 3.2	11.9 ± 2.2
SPC-IGFIR-Akt2^−/−^ (8 months)	100%	Not determined	29.0 ± 5.2^b^

a *p* < 0.05 compared to 9 month SPC-IGFIR

b *p* < 0.05 compared to 8 month SPC-IGFIR

**Figure 2:**
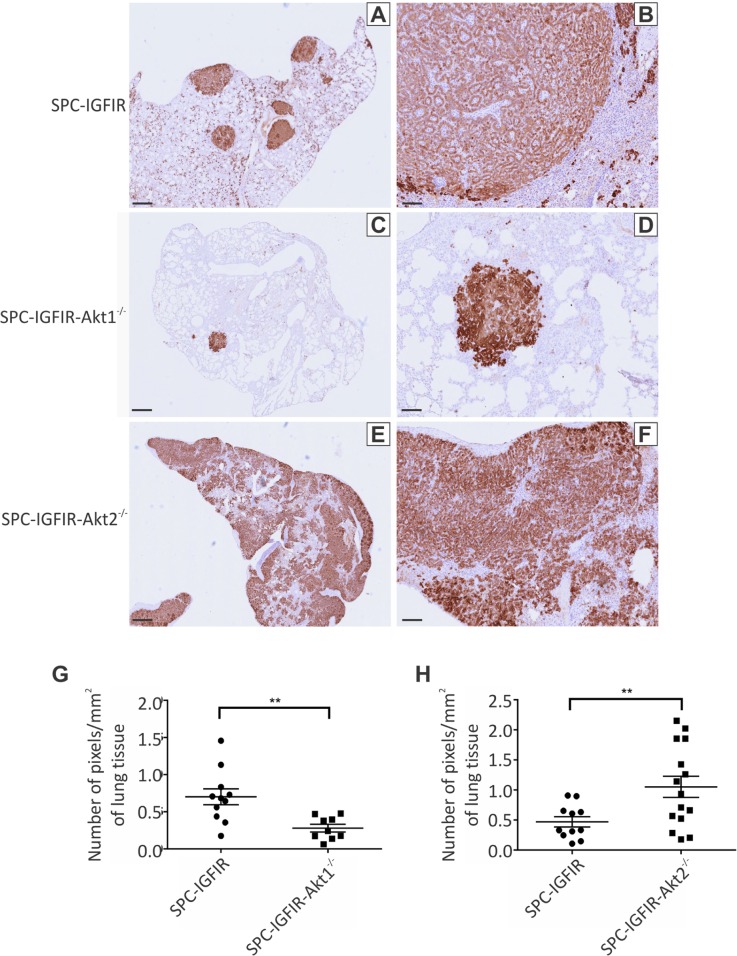
Tumor burden is increased in mice lacking *Akt2* but decreased in mice lacking *Akt1* Lungs from SPC-IGFIR **A, B.** SPC-IGFIR-*Akt1^−/−^* **C, D.** and SPC-IGFIR-*Akt2^−/−^* **E, F.** mice were stained using an anti-human IGF-IR antibody to determine transgenic IGF-IR expression. Images were obtained using ScanScope digital scanner and quantified using Aperio ImageScope software. Tumor burden was quantified using immunohistochemical analysis of transgenic IGF-IR expression in the lungs of SPC-IGFIR-*Akt1^−/−^* (*n* = 9) and SPC-IGFIR (*n* = 10) following 9 months of treatment with doxycycline **G.** and in SPC-IGFIR-*Akt2^−/−^* (*n* = 12) and SPC-IGFIR (*n* = 12) mice following 8 months of treatment with doxycycline **H.** Data is expressed as a ratio of the number of positive staining pixels to total area of lung tissue in mm^2^ and mean and SEM is depicted for each group. **p < 0.01. Error bars in A, C, E, 250 μm and B, D, F 100 μm.

The histological sections suggested that tumor burden was greatest in the SPC-IGFIR-*Akt2^−/−^* mice and lowest in the SPC-IGFIR-*Akt1^−/−^* mice. We took advantage of high IGF-IR expression in the tumor cells (Figure 2A–2F) and quantified tumor burden following IGF-IR immunohistochemistry as the number of positive pixels per total lung area (mm^2^). SPC-IGFIR-*Akt1^−/−^* mice (*n* = 9) had significantly reduced tumor burden compared to SPC-IGFIR mice (*n* = 10) after 9 months of IGF-IR overexpression (Figure 2G, Table 1) while SPC-IGFIR-*Akt2^−/−^* mice (*n* = 12) had significantly increased tumor burden compared to SPC-IGFIR mice (*n* = 12) after 8 months of IGF-IR overexpression (Figure 2H, Table 1).

Histology was performed on sections from major tissues including the liver, kidneys, pancreas, and brain. There were no obvious metastatic lesions observed in any of the tissues from any of the genotypes (data not shown).

### Loss of *Akt2* does not alter AKT or ERK1/2 activation

**Figure 3:**
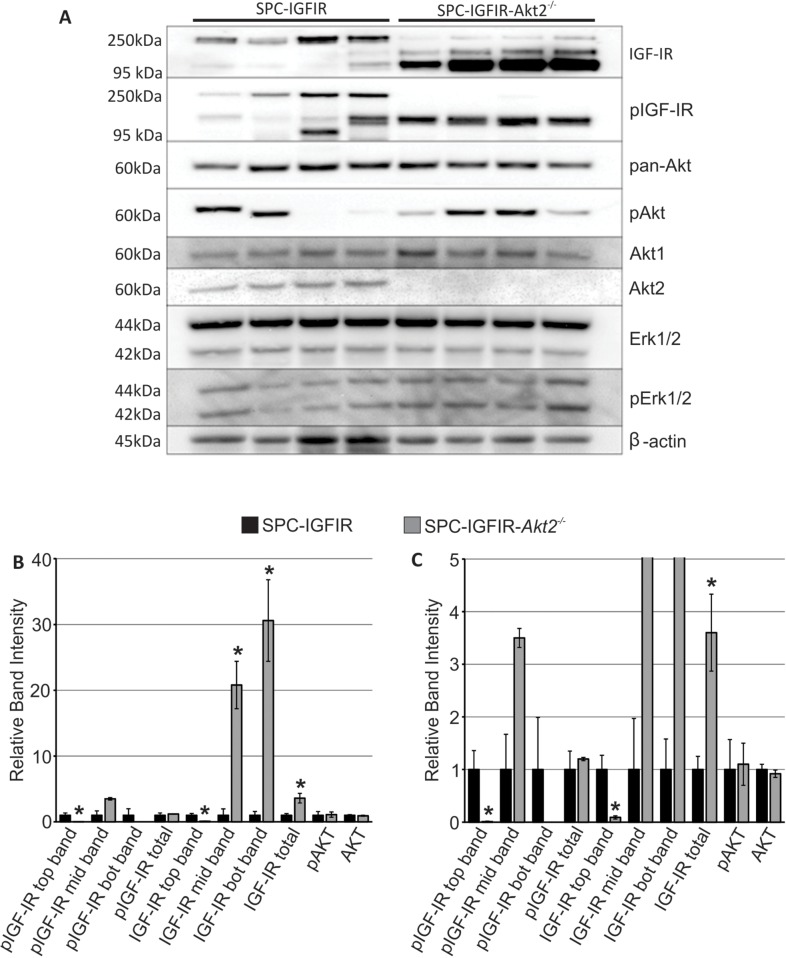
Tumors from SPC-IGFIR mice and SPC-IGFIR-*Akt2^−/−^* displayed difference in IGF-IR levels **A.** Western blot analysis of protein isolated from tumors from SPC-IGFIR and SPC-IGFIR-*Akt2^−/−^* mice. β-actin was used as a loading control. **B, C.** Quantification of the top, middle (mid) and bottom (bot) bands detected by antibodies against IGF-IR and phosphorylated IGF-IR as well as quantification of phosphorylated AKT and total AKT in SPC-IGFIR (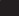) and SPC-IGFIR-*Akt2*^−/−^ (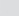) mice. Panel B shows the entire scale of relative band intensity while panel C highlight relative band intensities between 0–5, *p < 0.05.

Protein was isolated from lung tumors of SPC-IGFIR and SPC-IGFIR-*Akt2^−/−^* mice and analyzed by western blotting (Figure 3A). Due to the small number/size of tumors in the SPC-IGFIR-*Akt1^−/−^* mice there was insufficient tissue for protein isolation. As shown in Figure 3A, AKT2 protein was detected in SPC-IGFIR but not SPC-IGFIR-*Akt2^−/−^* tumors. In addition, the levels of AKT1 were similar in SPC-IGFIR and SPC-IGF-IR-*Akt2^−/−^* tumors suggesting that there was no compensatory increase in AKT1 in tumors lacking *Akt2* (AKT3 protein could not be detected; data not shown). Quantitative RT-PCR confirmed that neither *Akt1* nor *Akt3* mRNA expression was elevated to compensate for the loss of *Akt2* (data not shown). Similarly, there was no significant increase in *Akt2* or *Akt3* mRNA in the SPC-IGFIR-*Akt1^−/−^* tumors (data not shown).

The banding pattern for IGF-IR and phosphorylated IGF-IR was significantly different between SPC-IGFIR and SPC-IGFIR-*Akt2*^−/−^ tumors. Quantification of the western blots revealed that SPC-IGFIR-*Akt2*^−/−^ tumors had significantly lower levels of the highest molecular weight form of IGF-IR and the highest molecular weight version of phosphorylated IGF-IR while having significantly increased levels of the middle and lowest molecular weight form of IGF-IR as well as the total level of IGF-IR (Figure 3B, 3C). Although the levels of the 3 molecular weight bands detected varied between SPC-IGFIR and SPC-IGFIR-*Akt2*^−/−^ tumors, total phosphorylated IGF-IR was not significantly different between the two tumor types (Figure 3B, 3C). Similarly, the average levels of phosphorylated AKT did not vary significantly between tumor types despite considerable variation between samples (Figure 3B, 3C). Phosphorylated ERK1/2 and total ERK1/2 were not quantified as there were no apparent differences in the levels of these proteins between SCP-IGFIR and SPC-IGFIR-*Akt2*^−/−^ tumors.

### SPC-IGFIR and SPC-IGFIR-*Akt2*^−/−^ tumors express genes associated with human lung adenocarcinoma

To better characterize the SPC-IGFIR and SPC-IGFIR-*Akt2^−/−^* tumors RNA-seq was performed on RNA isolated from SPC-IGFIR tumors, SPC-IGFIR-*Akt2^−/−^* tumors and normal lung tissue (isolated tumors from SPC-IGFIR-*Akt1^−/−^* lungs were too small to obtain sufficient material for RNA-seq). Hierarchical clustering of samples based on correlation distance was performed to determine the similarity between samples and as expected, the tumor samples formed a distinct cluster from the normal lung tissue and 3 of 4 SPC-IGFIR-*Akt2^−/−^* tumors formed a distinct cluster from SPC-IGFIR tumors (Supplementary Figure S1).

**Figure 4:**
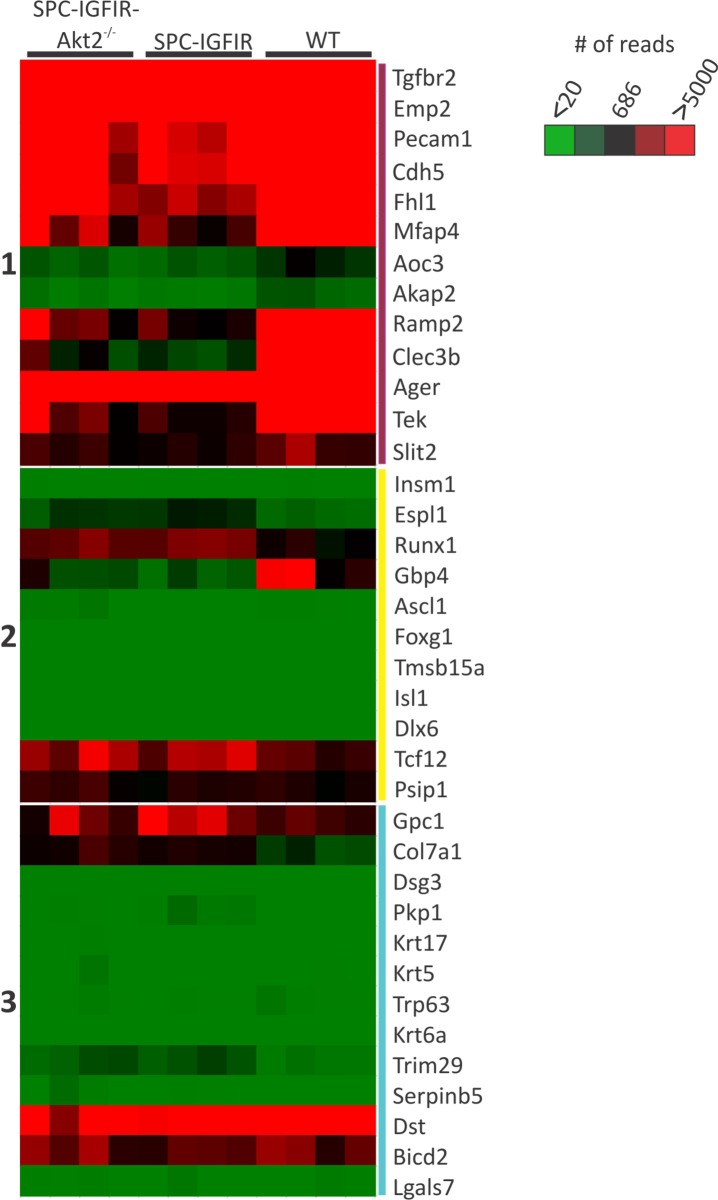
Lung tumors from SPC-IGFIR and SPC-IGFIR-*Akt2^−/−^* mice express high levels of genes associated with normal human lung tissue and human lung adenocarcinoma A heat map representing expression of genes from wild type (WT) murine lung, lung tumors from SPC-IGFIR mice (SPC-IGFIR) or lung tumors from SPC-IGFIR-*Akt2^−/−^* mice. Expression levels were determined from read counts from the RNA sequencing data. The median read count of the genes used was 686 and this value was set as zero (black). Genes with read counts > 686 were displayed with different shades of red while gene with read counts < 686 were displayed with different shades of green. Genes in group 1 are highly expressed in human normal lung and adenocarcinomas while genes in group 2 and group 3 are highly expressed in human carcinoid and squamous cell tumors, respectively.

The gene expression pattern of our murine tumors were then compared to human lung cancers. Bhattacharjee et al [50] evaluated gene expression profiles of different human lung cancers and identified gene clusters expressed at high levels that distinguished different human lung cancer subtypes. Using these gene signatures we found that our normal lung tissue expressed high levels of genes associated with normal human lung tissue (Figure 4, gene cluster 1). Most of the human lung adenocarcinomas expressed genes associated with the normal human lung tissue and not squamous cell, small cell or carcinoid tumors [50]. Similarly, both the SPC-IGFIR and SPC-IGFIR-*Akt2^−/−^* lung tumors expressed high levels of genes that were highly expressed in normal human lung tissue and adenocarcinomas (gene cluster 1) and not genes highly expressed by small cell and carcinoid tumors (cluster 2) or squamous cell tumors (cluster 3) suggesting that murine tumors are adenocarcinomas (Figure 4 and [50]).

### RNA sequencing identified gene and transcripts differentially expressed in SPC-IGFIR-*Akt2*^−/−^ tumors

Analysis of the RNA sequencing data revealed 112 genes were differentially expressed at least 2-fold (deseq adjusted *p*-value < 0.05) between SPC-IGFIR-*Akt2^−/−^* tumors and SPC-IGFIR tumors. The list of the top 20 up- and down-regulated genes based on fold expression differences in SPC-IGFIR-*Akt2^−/−^* tumors compared to SPC-IGFIR tumors are presented in Supplementary Table S1.

At the transcript level, 458 transcripts were differentially expressed at least 2-fold (q-value < 0.05) between SPC-IGFIR-*Akt2^−/−^* tumors and SPC-IGFIR tumors. The list of the top 20 up- and down-regulated transcripts based on fold expression differences in SPC-IGFIR-*Akt2^−/−^* tumors compared to SPC-IGFIR tumors are presented in Supplementary Table S2.

Ingenuity Pathway Analysis (IPA) software (Qiagen Redwood City, Redwood City, CA) was then used to identify pathways differentially regulated between SPC-IGFIR-*Akt2^−/−^* and SPC-IGFIR tumors. The same cutoffs were used for this analysis (at least 2-fold change in expression and *q*-value < 0.05 for transcripts and deseq adjusted *p*-value < 0.05 for genes). IPA software analyses only includes gene and transcript IDs that can be mapped to known genes in their database and this resulted in the inclusion of 88 genes and 387 transcripts for analysis. The top 3 diseases and disorders as well as the top 3 molecular and cellular functions for transcripts and genes are presented in Table 2. Supplementary Table S3 shows the pathways predicted to be significantly up or down regulated (Z-scores ≥ 2 or ≤-2) in the SPC-IGFIR-*Akt2^−/−^* tumors compared to the SPC-IGFIR tumors

**Table 2:** Diseases and Disorders or Molecular and Cellular Functions Identified by IPA Software Based on Transcripts and Genes

*TRANSCRIPTS*		
Diseases and Disorders	*p*-value range	# of molecules
Cancer	5.4×10^−4^ – 2.6×10^−12^	317
Organismal Injury and Abnormalities	5.4×10^−4^ – 2.6×10^−12^	319
Gastrointestinal Disease	4.7×10^−4^ – 2.8×10^−12^	137
**Molecular and Cellular Functions**		
Cellular development	5.5×10^−4^ – 1.7×10^−13^	150
Cellular movement	5.3×10^−4^ – 4.3×10^−12^	113
Cell growth and proliferation	5.4×10^−4^ – 3.5×10^−10^	158
***GENES***		
**Diseases and Disorders**	***p*-value range**	**# of molecules**
Inflammatory Disease	1.6×10^−2^ – 1.3×10^−6^	14
Inflammatory Response	2.0×10^−2^ – 1.3×10^−6^	29
Organismal Injury and Abnormalities	2.0×10^−2^ – 1.3×10^−6^	31
**Molecular and Cellular Functions**		
Cell-to-Cell Signaling and Interaction	2.0×10^−2^ – 5.9×10^−6^	31
Protein Synthesis	1.7×10^−2^ – 1.1×10^−5^	16
Cellular Development	2.0×10^−2^ – 1.4×10^−5^	33

**Figure 5:**
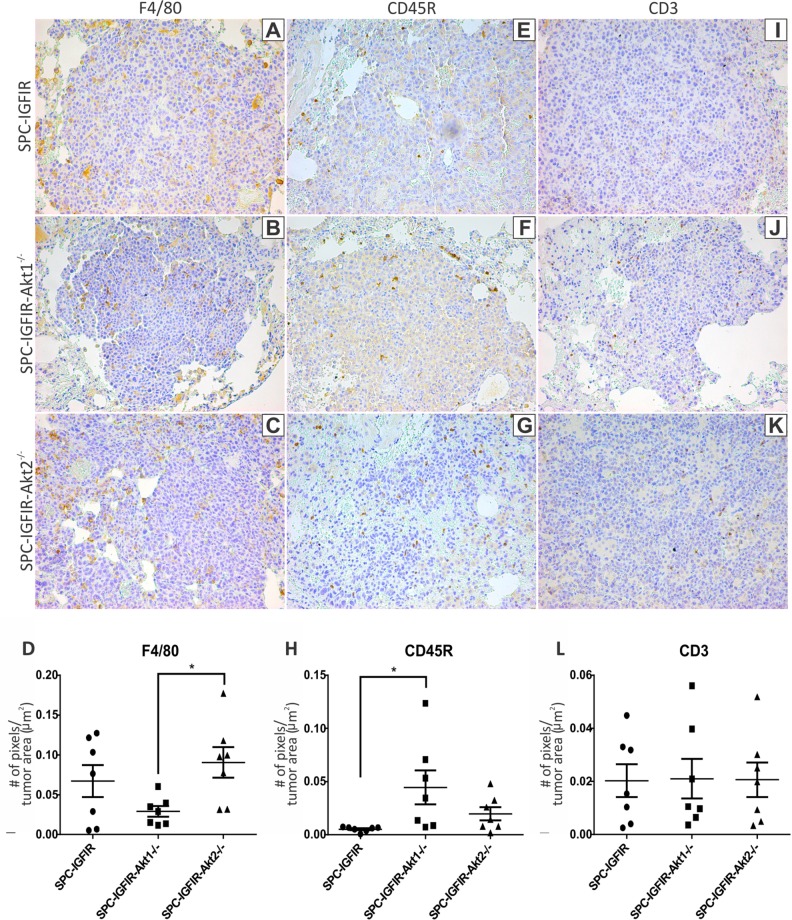
Lungs from SPC-IGFIR (A, E, I) SPC-IGFIR-*Akt1^−/−^* **(B, F, J).** and SPC-IGFIR-*Akt2^−/−^* **(C, G, K)**, mice were stained using antibodies specific for markers of macrophages (F4/80) **A-D.** B-lymphocytes (CD45R) **E-H.** and T-lymphocytes (CD3) **I-L.** Quantitation of the staining was determined using Aperio ImageScope software. Data is expressed as a ratio of the number of positive staining pixels to total area of lung tissue in μm^2^ **(D, H, L)** *p < 0.05.

Since the list of differentially expressed transcripts contained a large number of immunoglobulin related genes and the two top diseases/disorders identified by differentially expressed genes were associated with inflammation, immunohistochemistry was performed using a macrophage marker (F4/80; Figure 5A–5D), a B-lymphocyte marker (CD45R; Figure 5E–5H) and a T-lymphocyte marker (CD3; Figure 5I–5L). There were no significant differences in the number of macrophages, B-lymphocytes or T-lymphocytes between the SPC-IGFIR and SPC-IGFIR-*Akt2^−/−^* tumors (Figure 5D, 5H, 5L). There were however significant differences in the number of macrophages between SPC-IGFIR-*Akt2^−/−^* and SPC-IGFIR-*Akt1^−/−^* tumors (Figure 5D) and the number of B-lymphocytes between SPC-IGFIR-*Akt1^−/−^* and SPC-IGFIR tumors (Figure 5H).

The RNA-seq data was also mined for differences in gene expression that would indicate the presence or involvement of tumor associated macrophages (TAMs) (Supplementary Table S4) based on the study by Quatromoni and Eruslanov [51]. There were no significant differences in any of the genes suggesting that TAMs were unlikely to contribute to the differences in lung tumor development in SPC-IGFIR-*Akt2*^−/−^ mice compared to SPC-IGFIR mice.

### Transcripts potentially associated with lung tumorigenesis are differentially expressed in SPC-IGFIR-*Akt2*^−/−^ tumors

Nine transcripts were selected from the list of differentially expressed transcripts (SPC-IGFIR-*Akt2^−/−^* vs SPC-IGFIR) based on three criteria, (i) a fold change of at least 2, (ii) a *p*-value < 0.0002, and (iii) the gene had previously been implicated in human lung cancer (Table 3). Quantitative RT-PCR was used to confirm the changes in gene expression between SPC-IGFIR-*Akt2^−/−^* and SPC-IGFIR tumors and as shown in Table 3 all 9 genes showed similar patterns of expression in both analyses. For example, RNA sequencing found that *Bpifa1* expression was elevated ∼384-fold in SPC-IGFIR-*Akt2^−/−^* tumors compared to SPC-IGFIR tumors while qRT-PCR found a ∼279-fold increase in *Bpifa1* expression in SPC-IGFIR-*Akt2^−/−^* tumors compared to SPC-IGFIR tumors. Quantitative RT-PCR was also used to evaluate the expression of these 9 genes in SPC-IGFIR-*Akt1^−/−^* tumors relative to SPC-IGFIR tumors. Five of the genes (*Actc1, Bpifa1, Mmp2, Ntrk2, and Scgb3a2*) were differentially expressed in SPC-IGFIR-*Akt2^−/−^* tumors compared to SPC-IGFIR tumors but not SPC-IGFIR-*Akt1^−/−^* tumors compared to SPC-IGFIR tumors (Table 3) suggesting that these genes are specifically regulated in the SPC-IGFIR-*Akt2^−/−^* tumors and may contribute to the enhanced lung tumorigenesis observed in the SPC-IGFIR-*Akt2^−/−^* mice.

**Table 3:** Relative transcript expression of genes associated with lung cancer

Gene	Fold Change in Gene Expression in SPC-IGFIR-*Akt2^−/−^* Tumors Relative to SPC-IGFIR Tumors using RNA-seq	Fold Change in Gene Expression in SPC-IGFIR-*Akt2^−/−^* Tumors Relative to SPC-IGFIR Tumors using qRT-PCR	Fold Change in Gene Expression in SPC-IGFIR-*Akt1^−/−^* Tumors Relative to SPC-IGFIR Tumors using qRT-PCR
*Actc1*	7.8	11.3	1.1
*Bpifa1*	384.5	279.2	−1.3
*Ccl5*	6.0	1.6	3.4
*Crym*	−6.7	5.5	−2.5
*Cxcl12*	2.3	1.9	1.1
*Mdk*	3.3	4.1	2.5
*Mmp2*	2.4	2.1	−1.3
*Ntrk2*	4.0	4.8	1.0
*Scgb3a2*	5.8	6.2	1.0

### Human lung cancer cell lines are more sensitive to an AKT1 inhibitor than a pan-AKT inhibitor

**Figure 6:**
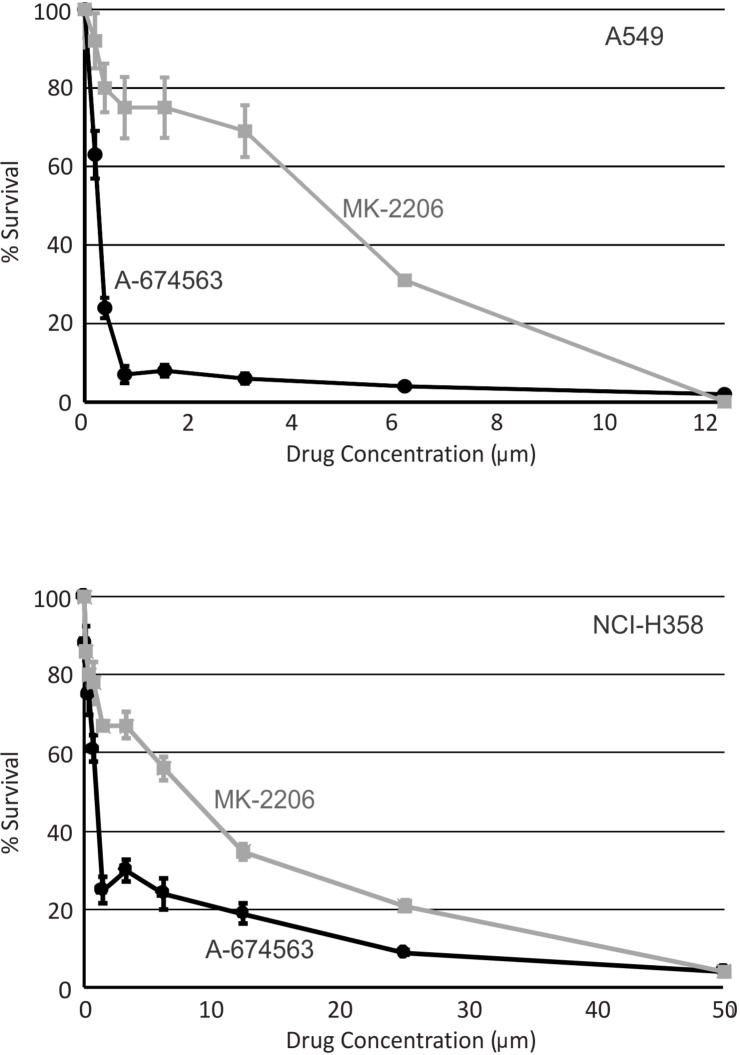
An AKT1 inhibitor is a more potent inhibitor of human lung cancer cell survival than a pan-AKT inhibitor Cell survival was determined using a WST-1 assay in the human lung cancer cells A549 **A.** and NCI-H358 **B.** following treatment with either an AKT1 specific inhibitor, A-674563 (black circle and line) or a pan-AKT inhibitor MK-2206 (gray square and line). *p < 0.05.

The data from our mouse model suggests that inhibition of AKT1 should suppress lung tumor growth while inhibition of AKT2 may have the opposite effect. Therefore, a selective AKT1 inhibitor may be a superior therapeutic strategy compared to inhibiting all AKT isoforms. The efficacy of the AKT1 specific inhibitor, A-674563, and the pan-AKT inhibitor, MK-2206, was evaluated *in vitro* using two human NSCLC cell lines. A549 and NCI-H358 cell lines were both established from male patients with NSCLC and both cell lines harbor *K-ras* mutations (http://atcc.org). *K-ras* is frequently mutated in lung adenocarcinoma with a prevalence of ∼25% reported [52]. A549 cells also have a mutation in *Cdnk2a* (http://atcc.org) while NCI-H358 cells harbor a homozygous deletion of *p53* [53]. As shown in Figure 6, A-674563 was a more potent inhibitor of cell survival than MK-2206 in both A549 (Figure 6A) and NCI-H358 (Figure 6B) cell lines. Cell counts using tyrpan blue exclusion confirmed these findings (data not shown).

## DISCUSSION

AKT is frequently implicated as an important mediator in the growth and development of many types of cancer, including lung cancer [8, 12]. Although AKT isoforms are only mutated in a small percentage of human lung cancers, AKT signaling is downstream of a number of oncogenes including PI3K, EGFR and HER2/EGFR2 [54–59]. Based on these observations, clinical trials have been initiated to evaluate the efficacy of pan-AKT inhibitors on NSCLC (http://www.cancer.gov/clinicaltrials). The selection of a pan-AKT inhibitor assumes that all 3 AKT isoforms promote tumor formation and progression, however, data from this current study and other studies suggest that AKT1 and AKT2 have opposite effects on lung tumorigenesis.

SPC-IGFIR transgenic mice express elevated levels of an *Igf1r* transgene in type II alveolar cells through the use of a doxycycline inducible, surfactant protein C (SPC) promoter system [48]. The *Igf1r* transgene is only expressed lung type II alveolar cells when the animals are provided with food or water supplemented with doxycycline which allows for transgene induction once lung development has completed [48]. In this study *Igf1r* transgene was induced by feeding mice rodent chow supplemented with 2g of doxycycline per kilogram of rodent chow beginning at 21 days of age. Previous characterization of SPC-IGFIR transgenic mice revealed that this approach induces lung tumor development in 100% of the mice by 9 months of age [48].

The SPC-IGFIR transgenic mouse model is relevant to lung cancer since IGF-IR is highly expressed in ∼95% of human SCLCs and ∼80% of NSCLCs [60–62] and high levels of *Igf1r* mRNA have been significantly associated with reduced overall survival and disease-free survival in NSCLC [63, 64]. The *in vitro* manipulation of human lung tumor cells also supports the idea that *Igf1r* is a critical regulator of lung tumorigenesis since targeting the IGF-IR protein inhibits lung tumor cell proliferation and sensitizes lung cancer cells to chemotherapy and radiotherapy [65–71]. In addition to conventional therapies, IGF-IR expression may also regulate the response of lung cancer to targeted therapies. For example, the IGF-IR has been implicated in mediating resistance to histone deacetylases inhibitors [72], anaplastic lymphoma receptor tyrosine kinase (ALK) inhibitors [73] and epidermal growth factor receptor (EGFR) inhibitors [74–76]. Moreover, a number of IGF-IR inhibitors are currently being evaluated in clinical trials for the treatment of lung cancer (http://clinicaltrials.gov).

Using our transgenic mouse model of IGF-IR driven lung cancer [48, 77], we found that loss of *Akt1* suppressed while loss of *Akt2* augmented, lung tumor development. These findings are similar to those of Linnerth-Petrik et al. [42] who also found that loss of *Akt1* inhibited while loss of *Akt2* increased tumor development in a virally induced mouse model of lung tumorigenesis. In mice exposed to the lung carcinogen (NNK) loss of *Akt1* or *Akt2* decreased lung tumor multiplicity but did not decrease lung tumor volume while loss of *Akt1* but not *Akt2* in *K-ras* transgenic mice significantly decreased tumor multiplicity and tumor volume [41]. Another lung cancer carcinogen, urethane, was evaluated by Hollander et al. [41] but only in *Akt2^−/−^* mice and it was observed that loss of *Akt2* increased tumor multiplicity and tumor volume following urethane exposure. Taken together, these findings demonstrate that loss of *Akt1* consistently suppresses lung tumorigenesis independent of the initiating event while loss of *Akt2* can promote lung tumorigenesis in response to some, but not all lung tumor initiating events.

The exact mechanisms through which loss of *Akt2* promotes, while loss of *Akt1* inhibits, lung tumorigenesis remains unclear. Unlike Linnerth-Petrik et al [42] we did not observe a significant change in proliferation (PCNA immunohistochemistry; data not shown) or apoptosis (cleaved caspase 3 immunohistochemistry; data not shown) in lung tumors from either SPC-IGFIR-*Akt1^−/−^* or SPC-IGFIR-*Akt2^−/−^* mice. In our model, it is possible that AKT1 sensitized lung epithelial cells to transformation by IGF-IR. This theory is supported by observations that AKT1 was required for transformation of immortalized mouse fibroblasts expressing mutant *K-ras* and a dominant negative *p53* construct [41]. In addition, Nomura et al [78] reported that expression of a dominant negative AKT1 prevented transformation, but did not impact proliferation, of a mouse epidermal cell line.

Western analysis of IGF-IR, AKT, ERK1/2 and the phosphorylated forms of these protein revealed considerable variation in the samples with respect to IGF-IR and AKT (ERK1/2 levels and phosphorylation were consistently expressed across all samples). The IGF-IR antibody used in this study detects the beta subunit of the IGF-IR and in the tumor samples detected 3 different molecular weight versions of IGF-IR. Although the molecular weights do not completely match up, these 3 bands presumably reflected the pro-IGFIR (200–250kDa), the alpha-beta IGF-IR precursor (∼180kDa) and the beta subunit of the IGF-IR (∼90kDa) [79, 80]. The western blots suggest differential processing of the IGF-IR in SPC-IGFIR and SPC-IGF-*Akt2*^−/−^ tumors however the physiologic relevance of this processing and the factors influencing processing remain unclear. A search of the literature could not find any manuscripts describing the ability of the AKT isoforms to modulate IGF-IR processing. It is interesting to note that total phosphorylated IGF-IR was similar in SPC-IGF-IR and SPC-IGF-*Akt2*^−/−^ tumors which may indicate similar levels of receptor activation. Phosphorylated AKT was also variable between different tumors however that average levels of phosphorylated AKT was not significantly different between SPC-IGFIR and SPC-IGF-*Akt2*^−/−^ tumors. The study by Linnerth-Petrik et al [42] which evaluated the impact of *Akt2* loss in a viral lung cancer model did not directly compare AKT phosphorylation in parental and *Akt2* null mice however, the levels of AKT phosphorylation were relatively consistent across tumors at different stages of development.

Our study is the first to perform RNA-seq analysis on murine lung tumors. RNA-seq analysis of SPC-IGFIR and SPC-IGFIR-*Akt2^−/−^* tumors showed that although the tumors clustered independently from normal lung tissue there was some overlap in gene expression between lung tumors with and without *Akt2* expression. We first used the expression profiling data to determine the human lung cancer subtype we were modeling in the SPC-IGFIR and SPC-IGFIR-*Akt2^−/−^* tumors and whether loss of *Akt2* altered the lung tumor subtype. Using the gene signatures identified by Bhattacharjee et al [50] we found that our normal murine lung tissue expressed high levels of genes associated with normal human lung tissue thus providing validation for the use of this signature. Both the SPC-IGFIR and SPC-IGFIR-*Akt2^−/−^* tumors expressed high levels of genes associated with normal human lung tissue and human lung adenocarcinomas and not genes associated with squamous cell carcinomas, small cell lung cancers or pulmonary carcinoids [50]. This analysis demonstrated that lung tumors induced by IGF-IR overexpression expressed markers consistent with adenocarcinomas whether or not AKT2 was present.

Further analysis of the RNA-seq data revealed a large number of immune system related transcripts. To determine whether alterations in immune cells were regulating lung tumorigenesis in our model the number of macrophages, T-lymphocytes and B-lymphocytes was examined via immunohistochemistry. No significant differences in the number of immune cells were observed between SPC-IGFIR and SPC-IGFIR-*Akt2^−/−^* tumors. It should be noted that immune cell number only provides one aspect of the immune response and does not provide information regarding the activity or function of the immune cells associated with tumors. Of the top 20 transcripts downregulated in SPC-IGFIR-*Akt2^−/−^* tumors compared to SPC-IGFIR tumors, 14 of these were associated with immunoglobulin genes. Therefore, it is possible that immunoglobulin production by B-lymphocytes is impaired in the SPC-IGFIR-*Akt2^−/−^* tumors. A Pubmed search did not reveal any manuscripts describing changes in *Igkv* or *Ighv* genes/transcripts in human lung cancer. One manuscript found that *Ighg1* was elevated in malignant mesothelioma [81] and *Ighg1* has been associated with immune evasion, proliferation and protection from apoptosis in prostate cancer [82, 83]. In our study *Ighg1* downregulation was associated with increased tumor burden and thus Ighg1 is not promoting tumorigenesis in the SPC-IGFIR-*Akt2^−/−^* mice.

The RNA-seq data was also mined for markers of tumor associated macrophages (TAMs). Macrophages can be broadly classify as M1 or M2 subtypes where M1 macrophages typically respond to bacterial infections and are thought to be involved in suppressing tumorigenesis while M2 macrophages can promote tumor development [51, 84]. Tumor derived cytokines such as IL-4, IL-10, IL-13, TGFβ and prostaglandin E2 can drive the production of M2 macrophages while IL-12 is produced by M1 macrophages [51, 85, 86]. We found no significant differences in the levels of any of these cytokines or enzymes involved in the generation of prostaglandin E2 in SPC-IGFIR tumors compared to SPC-IGFIR-*Akt2^−/−^* tumors (Supplementary Table S4). *Pdl1* expression was also evaluated since M2 macrophages express *Pdl1* [87] and like the cytokines, no significant differences were observed. Therefore, it is unlikely that tumor associated macrophages are driving the enhance lung tumorigenesis observed in the SPC-IGFIR-*Akt2^−/−^* mice.

It is also possible that alterations in glucose homeostasis contributed to the enhanced tumor burden observed in *Akt2* null mice. *Akt2* null mice are known to develop mild hyperglycemia and hyperinsulinemia [37, 38]. Hyperglycemia and hyperinsulinemia have been implicated in promoting the incidence of a number of tumors [88] and hyperglycemia has been associated with a poor prognosis in patients with lung cancer [10]. Moreover, one of the functions with a significant, positive z-score in the SPC-IGFIR-*Akt2^−/−^* tumors was carbohydrate metabolism. However, an increased tumor burden in *Akt2* null mice is not consistently seen in mouse models of lung cancer [41] or other tumors such as mammary tumors [89], which would be expected if hyperglycemia was the driving force behind accelerated tumorigenesis in *Akt2^−/−^* models. One of the more striking differences in the lung tumors that developed in the SPC-IGFIR-*Akt2^−/−^* mice was the diffuse tumor growth pattern. While the lung tumors of SPC-IGFIR and SPC-IGFIR-*Akt1^−/−^* mice typically grew as discrete nodules, the lung tumors in SPC-IGFIR-*Akt2^−/−^* mice rarely displayed a nodular tumor growth pattern. There are at least three possible explanations for the altered tumor growth pattern found in the SPC-IGFIR-*Akt2−/−* mice. First, it is possible that the tumor cells in SPC-IGFIR-*Akt2−/−* mice are more motile. Increased motility is often associated with epithelial to mesenchymal transition (EMT) in tumor cells. However, evaluation of a number of genes (Zeb1, Zeb2, Twist, vimentin, etc) associated with EMT did not support the idea that SPC-IGFIR-*Akt2−/−* tumor cells had undergone EMT (Supplementary Table S5). In addition, the expression of the major claudin genes (3, 4, 5, 7, and 18) which participate in cell-cell adhesion through their function as tight junction proteins [90] were not significantly different between SPC-IGFIR and SPC-IGFIR-*Akt2−/−* tumors. Cells undergoing EMT frequently express lower levels of tight junction genes. Even in lobes of lungs from SPC-IGFIR-*Akt2^−/−^* mice where tumor burden was not as extensive, nodular tumor formation was rare. Therefore it does not appear that tumors in SPC-IGFIR-*Akt2^−/−^* mice initiate as nodules followed by tumor cell dissemination via enhanced migration. The second possible explanation is that loss of *Akt2* resulted in an increased number of type II alveolar cells. No lung phenotype has been described in *Akt2^−/−^* mice and although RNA-seq was not performed on non-tumor bearing *Akt2^−/−^* lung tissue we did find a non-significant, 2.6-fold increase in *Sftpc* expression (a marker of type II alveolar cells) in SPC-IGFIR-*Akt2^−/−^* tumors compared to SPC-IGFIR tumors. One could argue that this increase in *Sftpc* simply reflects the increased tumor burden found in the SPC-IGFIR-*Akt2^−/−^* mice however RNA-seq comparing lungs from wild type mice to SPC-IGFIR lung tumors found a significant, 2.5-fold reduction in SPC-IGFIR tumors compared to normal lung tissue (unpublished observations). Therefore, *Sftpc* expression is unlikely to reflect tumor burden. Thus, the enhanced *Sftpc* expression found in the SPC-IGFIR-*Akt2^−/−^* tumors could be due to an increase in the number of type II alveolar resulting from the loss of *Akt2* or it could indicate that the tumor cells in the SPC-IGFIR-*Akt2^−/−^* mice are more differentiated and express higher levels of *Sftpc* than tumor cells in SPC-IGFIR mice. The final possibility is that loss of *Akt2* renders type II alveolar cells more susceptible to transformation. Given that tumor burden is higher in SPC-IGFIR-*Akt2^−/−^* and there is no significant difference in tumor cell proliferation, this suggests that the increased tumor burden resulted from an elevated number or transformation events. This idea is supported by the data of Linnerth-Petrik et al [42] who showed the number of individual lung tumors was significantly higher in *Akt2^−/−^* mice than *Akt1^−/−^* or control mice at early time points.

Focusing on the differentially expressed genes and transcripts that have previously been implicated in human lung cancer revealed some potential candidates that could contribute to the enhanced tumor development observed in SPC-IGFIR-*Akt2^−/−^* mice. *Bpifa1, Actc1, Scgb3a2, Ntrk2 and Mmp2* were all significantly upregulated in SPC-IGFIR-*Akt2^−/−^* but not SPC-IGFIR-*Akt1^−/−^* tumors compared to SPC-IGFIR tumors. *Bpifa1* (BPI Fold Containing Family A, Member 1), also known as *Lunx* or *Splunc1* in humans and *Plunc* in mice, is expressed in the upper airways and nasopharyngeal regions and is involved in regulating inflammatory responses to irritants and displays antibacterial properties against Gram-negative bacteria [91]. This gene has been associated with poor prognosis in lung cancer and shows promise as a lung cancer biomarker [92–97]. Zheng et al [98] have shown that overexpression of *Bpifa1* increases lung cancer cell proliferation and migration while *Bpifa1* silencing inhibited tumor growth, invasion and metastasis. *Scgb3a2* or secretoglobin 3A2 codes for a protein that is secreted by lung epithelial cells that plays a role in lung development [99] and has anti-inflammatory and antifibrotic activities [100]. Immunohistochemistry for SCGB3A2 revealed that this protein was expressed in 74% of primary lung tumors and was predominantly expressed in adenocarcinomas (86%) [101]. There was no association between SCGB3A2 and tumor differentiation, pathological stage or survival [101]. *Ntrk2* (neurotrophic tyrosine kinase, receptor, type 2), also known as tropomyosin-related kinase B (TrkB) plays a role in neural development [102]. This gene is a prognostic marker for a number of cancers including NSCLC [102–104]. In human lung adenocarcinoma cell lines and a mouse model of adenocarcinoma, *Ntrk2* promoted cell migration and tumor cell metastasis [105]. In addition, *Ntrk2* was identified as one of eighteen genes that could replace EGFR dependence in NSCLC and these genes replace EGFR dependence by stimulating EGFR-independent signaling through MEK-ERK and PI3K-AKT signaling [106]. *Mmp-2* is a member of the matrix metalloproteinase family. This family, and in particular *Mmp-2* and *Mmp-9*, have been implicated in a variety of cancers [107]. A meta-analysis performed in 2010 demonstrated that high expression of *Mmp-2* was marker of poor prognosis in NSCLC and in adenocarcinoma patients [108]. MMP-2 has been shown to regulate NSCLC migration and metastasis and genes, [109–112] micro-RNAs [113, 114] or chemicals [115–117] have been shown to mediate their anti-migratory effects through negatively regulating MMP-2 as well as other matrix metalloproteinases such as MMP-9. The only paper that has evaluated *Actc1* in lung cancer and this study showed that *Actc1* expression was elevated in the human lung cancer cell line NCI-460 following treatment with paclitaxel [118]. Function and regulation of these genes are areas currently being explored in our lab.

As the number of studies examining AKT isoform specific effects grow, it is becoming increasingly clear that AKT isoforms are not redundant in activity and have independent and differing physiologic roles. The clinical implications of AKT isoforms require consideration, especially in light of our findings that an AKT1 selective inhibitor significantly reduced survival in NSCLC cell lines harboring *K-ras* mutations to a greater extent the pan-AKT inhibitor, MK-2206. The nanomolar to micromolar IC_50_ values for A-674563 in both A549 and NCI-H358 cells is encouraging and suggests that even in tumor cells with *K-ras* mutations, these cells remain at least partially dependent on AKT for survival. The reduced sensitivity of NCI-H358 cells compared to A549 may suggest that cells harboring *p53* mutations may be less dependent on AKT signaling for survival. Therefore, both the genetic manipulation of *Akt1* in the SPC-IGFIR transgenic mice and the use of a selective AKT1 inhibitor in human lung cancer cell lines suggests that AKT1 is a critical regulator of lung adenocarcinoma development, particularly in tumor cells containing wild type *p53*. Currently only the MK-2206 is being evaluated in clinical trials and our data suggests that AKT1 selective inhibitors may warrant evaluation in clinical trials, at least for lung adenocarcinomas expressing wild type *p53*.

In summary, pre-clinical studies present compelling evidence that targeting AKT1 is an effective strategy to impair lung cancer development and future clinical trials on NSCLC may benefit by incorporating AKT1 inhibitors into their therapeutic strategy.

## MATERIALS AND METHODS

### Mice

SPC-IGFIR transgenic mice were previously described in Linnerth et al [48]. SPC-IGFIR mice were mated with *Akt1^−/−^* or *Akt2^−/−^* mice backcrossed into an FVB background [89] producing SPC-IGFIR-*Akt1^−/−^* and SPC-IGFIR-*Akt2^−/−^* mice (all mice were in FVB background). Mice were fed rodent-chow supplemented with doxycycline (2g/kg) ad libitum starting at 21 days of age to induce *Igf1r* transgene expression. Mice were maintained and cared for following the Canadian Council for Animal Care guidelines and ethical approval was provided by the Animal Care Committee at the University of Guelph.

Mice were euthanized following either 8 months (SPC-IGFIR-*Akt2^−/−^* and SPC-IGFIR controls) or 9 months (SPC-IGFIR-*Akt1^−/−^* and SPC-IGFIR controls) of treatment with doxycycline. A small amount of lung tumor or normal lung were isolated and flash frozen in liquid nitrogen. The remainder of the lung tissue, containing tumors, and other tissues including liver, kidney, spleen, and brain were fixed in 10% buffered formalin for 24 hours and embedded in paraffin. Additionally, normal lung tissue from wild-type mice which had been treated with doxycycline was collected as a control.

### Cells

A549 and NCI-H358 human lung cancer cells were purchased from American Type Culture Collection (ATCC, Manassas, VA). A549 and NCI-H358 cells were cultured in RPMI 1640 media (Life Technologies, Burlington, ON) supplemented with 10% FBS (Life Technology, Burlington, ON) and 1% antibiotic/antimycotic (Life Technologies, Burlington, ON). Cells were maintained at 37° C and 5% CO_2_.

### Histology and immunohistochemistry

Sections were deparaffinized, reyhydrated and were either stained with hematoxylin and eosin or continued with immunohistochemistry. Antigen retrieval was performed using 10 mM citrate buffer (pH 6.0) or TRIS-EDTA (10 mM Tris, 1 mM EDTA, pH 9.0; for CD3 antibody only). Tissues were blocked in 10% BSA in PBST (PBS containing 0.1% Triton-X). Tissues being stained for CD3 were additionally blocked in avidin (0.001% in PBS) then biotin (0.001% in PBS) (Sigma-Aldrich, Oakville, ON). Antibodies were diluted in 1% BSA in PBST and primary antibodies were used against human (transgene) IGF-IR (1:1000) (R&D Systems, Minneapolis, MN), CD3(1:500), CD45R(1:2000), F4/80(1:500) (AbD Serotec, Raleigh, NC), and proliferating cell nuclear antigen (PCNA) (1:100) (Santa Cruz Biotechnology, Dallas, TX) overnight at 4°C. Tissues were incubated in secondary anti-goat (1:200), anti-rat (1:500), or anti-rabbit (1:100) antibody (Sigma-Aldrich, Oakville, ON) then extravidin (1:50) (Sigma-Aldrich, Oakville, ON) for one hour each at room temperature then treated with Sigma Fast 3,3′-diaminobenzidine tablets (Sigma-Aldrich, Oakville, ON). Sections were counterstained with hematoxylin, dehydrated and mounted with Cytoseal XYL mounting media (Thermo Scientific, Waltham, MA).

Sections of lung tissue with immunohistochemistry staining for the transgenic IGF-IR were scanned with an Aperio Scanscope GL (Leica Biosystems, Concord, ON) slide scanner. Images of all other slides were captured using Nikon E600 microscope and Q-Capture software (Q-Imaging, Surrey, BC). Staining was quantified using Aperio ImageScope software (Leica Biosystems, Concord, ON). Transgenic IGF-IR was used as a marker for tumor burden, which was calculated for each mouse as the number of positive pixels per total area of lung tissue analyzed (mm^2^).

### Western blotting

Protein isolation and western blots were performed as previously described [119]. Primary antibodies were used against IGF-IRβ (1:1000), phospho-IGF-IR/IR (1:500), phospho-AKT (1:1000), pan-AKT (1:1000), AKT1 (1:1000), AKT2 (1:500) phospho-ERK1/2 (1:500), ERK (1:1000) and β-actin (1:5000) (Cell Signaling Technology, Danvers, MA) and against human (transgenic) IGF-IR (1:1000) (R&D Systems, Minneapolis MN) overnight at 4°C. Membranes were then incubated with either an anti-rabbit (1:2000) (Cell Signaling Technology, Danvers, MA) or anti-goat (1:2000) (Santa Cruz Biotechnology. Dallas, TX) secondary antibody for one hour at room temperature. Membranes were visualized using chemiluminescence substrate (PerkinElmer, Waltham, MA) and FluoroChem 8800 gel documentation system (Alpha Innotech - ProteinSimple, Toronto, ON).

### RNA isolation and QRT-PCR

RNA was isolated using the mirVana miRNA Isolation Kit (Ambion - Life Technologies, Burlington, ON) according to manufacturer protocol. RNA (250 ng) was reverse-transcribed using SuperScript II Reverse Transcriptase, 5x First-Strand buffer, DTT, RNaseOUT Recombinant Ribonuclease Inhibitor, Oligo(dT)12–18, and dNTP (Life Technologies, Burlington, ON). Gene expression was determined using qPCR reaction with Platinum SYBR Green qPCR SuperMix-UDG (Life Technologies, Burlington, ON) and performed on the CFX96 Real-time PCR Detection System (Bio-rad Laboratories, Mississauga, ON). Primers for qPCR were used for *Mmp2, CxCl12, Mdk, Ntrk2, Bpifa1, Actc1, Ptpr21, Scgb3a2, Ccl5, Crym, Hprt* (Bio-rad Laboratories, Mississauga, ON), and *Actb* (Origene Technologies, Rockville, MD). Relative quantification of gene expression using qPCR was determined using the ΔΔCq method normalizing with *Hprt* and *Actb* as reference genes using CFX-Manager 3.1 (Bio-rad Laboratories, Missisauga, ON).

### RNA sequencing

RNA sequencing and analysis was performed on tumor tissue from SPC-IGFIR and SPC-IGFIR-*Akt2^−/−^* mice as well as normal lung tissue from wild-type mice at the Genome Quebec Innovation Centre at McGill University using the Illumina Hiseq 2000/2500 sequencer. Reads were trimmed using Trimmomatic software [120] from the 3′ end to have a phred score of at least 30. Sequencing adapters were removed from the reads and only reads of at least 32 base pairs were used. Filtered reads were aligned to the MGSC_V37_ reference genome using tophat/bowtie software [121]. RNA-seq reads were aligned into transcripts and their abundance estimated using Cufflinks [122]. Additional unsupervised heirearchal clustering analysis was performed using Gene Cluster 3.0 and visualized with Java TreeView [123]. RNA Sequencing data was further analyzed with Ingenuity Pathway Analysis (IPA) software (QIAGEN, Redwood City, CA) using transcripts with at least 2 fold difference in expression and a minium *p*-value (significance) and *q*-value (false discovery rate) of 0.05.

### Cell survival assays

A549 and NCI-H358 cell survival was assessed using a WST-1 assay. Cells were seeded in 96-well tissue culture plates at a density of 1 × 10^3^ cells per well. After 24 hours DMSO or increasing concentrations of A-674563 (AKT1 inhibitor) or MK-2206 (pan-AKT inhibitor) (Selleck Chemicals LLC, Houston, TX) were added to the wells (DMSO concentration was maintained at 0.01% in all wells). Media and inhibitors were replaced every 24 hours and survival was assessed 72 hours after the initiation of drug treatment. Cell number was approximated by adding 10 μL of WST-1 reagent (Life Technologies, Burlington, ON) to each well and incubating at 37° C for 2 hours. Optical density of each well was determined at 450 nm using an EL-800 microplate reader (BioTek Winooski, VT). Survival curves were generated relative to the DMSO control.

### Statistics

Statistical analysis was performed using Graphpad Prism 6 (Graphpad Software, Inc., La Jolla, CA). Means were compared using a Student's *T*-test or a one way ANOVA followed by a post-hoc Tukey's Test. Error is represented by standard error of measurement (SEM). Statistical significance is noted as *p* < 0.05.

## SUPPLEMENTARY FIGURES AND TABLES


