# Supplementary material for: Unique roles of Akt1 and Akt2 in IGF-IR mediated lung tumorigenesis
Source: Oncotarget. 2015 Dec 7;7(3):3297–316. doi: 10.18632/oncotarget.6489 (PMC4823107; doi:10.18632/oncotarget.6489)
Supplement: Supplementary file 1 [file oncotarget-07-3297-s001.pdf]

SUPPLEMENTARY FIGURE AND TABLES

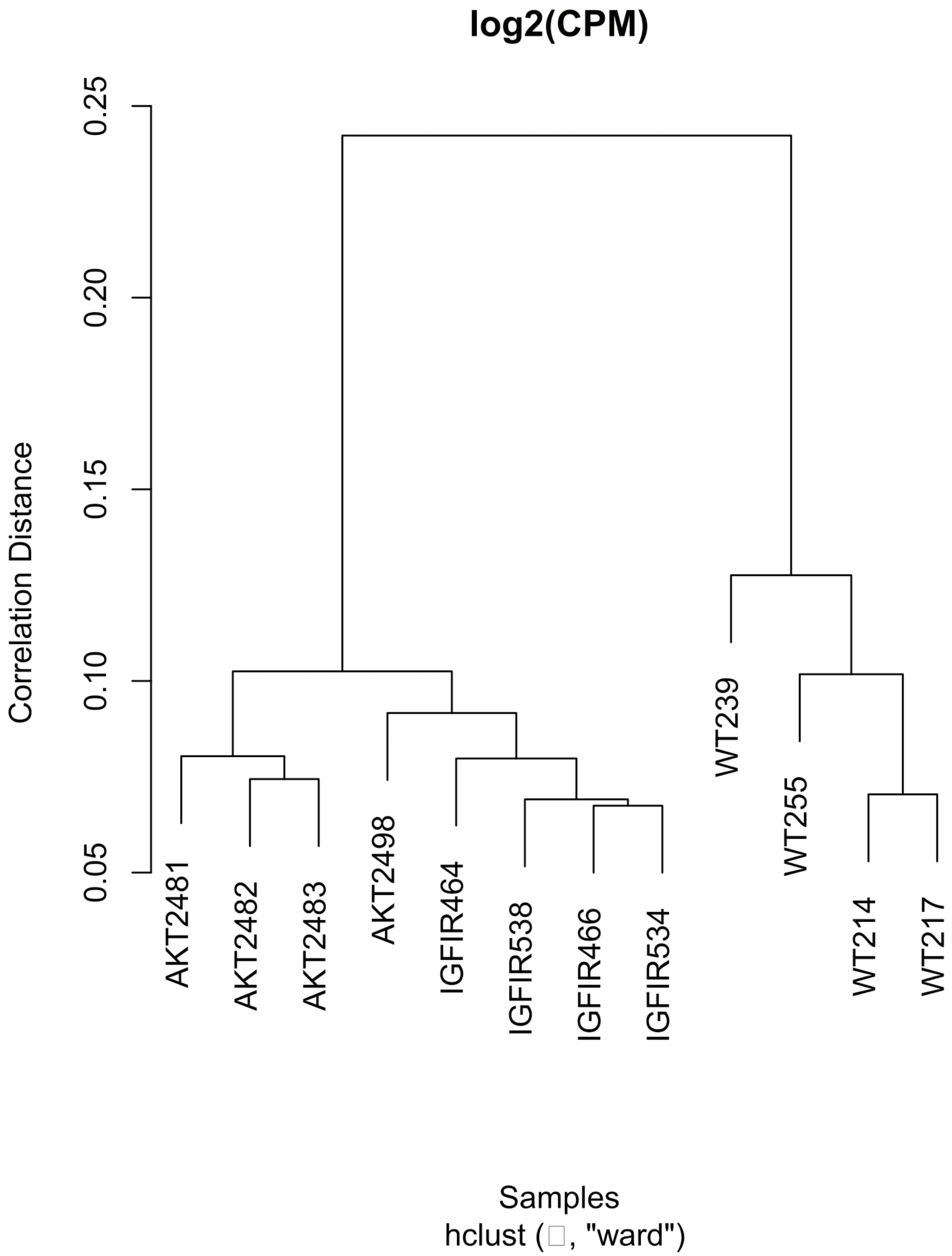

Supplementary Figure S1: Hierachical clustering of samples based on the correlation distance (Ward approach), $\log_2$ (RPKM).

Supplementary Table S1: Top 20 Genes Differentially Regulated in SPC-IGFIR-*Akt2*$^{-/-}$ Tumors Compared to SPC-IGFIR Tumors

Genes Upregulated in SPC-IGFIR-*Akt2*$^{-/-}$ Tumors			
**Gene ID**	**Gene Symbol**	**Fold Change**	**Deseq Adjusted *p*-val**
ENSMUSG00000057836	Xlr3a	38.8	$1.4x10^{-2}$
ENSMUSG00000054385	Ceacam2	34.8	$9.1x10^{-4}$
ENSMUSG00000043192	Gm1840	16.8	$3.9x10^{-3}$
ENSMUSG00000044006	Cilp2	14.4	$1.0x10^{-3}$
ENSMUSG00000021587	Pcsk1	13.2	$9.1x10^{-3}$
ENSMUSG00000036231	Agr3	12.0	$3.4x10^{-2}$
ENSMUSG00000060962	Dmkn	10.6	$4.2x10^{-3}$
ENSMUSG00000047420	Fam180a	9.0	$1.2x10^{-2}$
ENSMUSG00000030114	Klrg1	7.7	$4.4x10^{-6}$
ENSMUSG00000073125	Xlr3b	5.9	$1.9x10^{-2}$
ENSMUSG00000053550	Shisa7	5.7	$1.7x10^{-3}$
ENSMUSG00000026011	Ctla4	5.4	$3.1x10^{-3}$
ENSMUSG00000034295	Fhod3	4.6	$1.6x10^{-2}$
ENSMUSG00000068794	Col28a1	4.1	$6.7x10^{-4}$
ENSMUSG00000048040	Arxes2	4.1	$7.4x10^{-4}$
ENSMUSG00000029075	Tnfrsf4	4.0	$4.6x10^{-2}$
ENSMUSG00000059327	Eda	3.9	$1.0x10^{-2}$
ENSMUSG00000018566	Slc2a4	3.6	$1.4x10^{-2}$
ENSMUSG00000039084	Chad	3.6	$9.0x10^{-5}$
ENSMUSG00000022357	Klhl38	3.5	$4.3x10^{-3}$
**Genes Downregulated in SPC-IGFIR-Akt2$^{-/-}$ Tumors**			
Gene ID	Gene Symbol	Fold Change	Deseq Adjusted Pval
ENSMUSG00000085627	Gm11222	140.2	$1.4x10^{-2}$
ENSMUSG00000052551	Adarb2	16.1	$3.9x10^{-3}$
ENSMUSG00000092624	Gm3654	14.7	$1.4x10^{-2}$
ENSMUSG00000092312	Zfp419	13.2	$6.7x10^{-3}$
ENSMUSG00000087267	4933427J07Rik	9.6	$1.0x10^{-2}$
ENSMUSG00000006342	Susd2	9.3	$1.1x10^{-3}$
ENSMUSG00000062017	Abca14	6.8	$8.8x10^{-3}$
ENSMUSG00000030905	Crym	6.8	$1.1x10^{-12}$
ENSMUSG00000074417	Gm14548	6.7	$9.0x10^{-3}$
ENSMUSG00000008153	Clstn3	5.5	$1.6x10^{-3}$
ENSMUSG00000049939	Lrrc4	4.1	$7.4x10^{-5}$
ENSMUSG00000033253	Szt2	3.9	$4.4x10^{-6}$

(*Continued*)

**Genes Upregulated in SPC-IGFIR-*Akt2*$^{-/-}$ Tumors**			
**Gene ID**	**Gene Symbol**	**Fold Change**	**Deseq Adjusted *p*-val**
ENSMUSG00000060180	Myh13	3.5	$1.8x10^{-3}$
ENSMUSG00000029868	Trpv6	3.5	$2.0x10^{-2}$
ENSMUSG00000079620	Muc4	3.5	$2.2x10^{-2}$
ENSMUSG00000042268	Slc26a9	3.3	$1.4x10^{-3}$
ENSMUSG00000026764	Kif5c	3.3	$6.1x10^{-3}$
ENSMUSG00000059674	Cdh24	3.0	$7.7x10^{-}$
ENSMUSG00000027070	Lrp2	3.0	$6.7x10^{-7}$

Supplementary Table S2: Top 20 Transcripts Differentially Regulated in SPC-IGFIR-*Akt2*$^{-/-}$ Tumors Compared to SPC-IGFIR Tumors

**Transcripts Upregulated in SPC-IGFIR-*Akt2*$^{-/-}$ Tumors**					
**Test ID**	**Gene ID**	**Gene Name**	**FoldChange**	***p*-val**	**q-val**
ENSMUST00000028985	ENSMUSG00000027483	Bpifa1	384.5	$5.0x10^{-5}$	$7.1x10^{-3}$
ENSMUST00000035077	ENSMUSG00000032496	Ltf	25.3	$5.0x10^{-5}$	$7.1x10^{-3}$
ENSMUST00000005685	ENSMUSG00000005547	Cyp2a5	21.2	$5.0x10^{-5}$	$7.1x10^{-3}$
ENSMUST00000057831	ENSMUSG00000044006	Cilp2	19.7	$1.5x10^{-3}$	$1.7x10^{-2}$
ENSMUST00000064862	ENSMUSG00000054385	Ceacam2	14.2	$1.0x10^{-4}$	$1.2x10^{-2}$
ENSMUST00000043873	ENSMUSG00000064057	Scgb3a1	13.7	$5.0x10^{-5}$	$7.1x10^{-3}$
ENSMUST00000060574	ENSMUSG00000047228	BC048546	12.8	$5.0x10^{-5}$	$7.1x10^{-3}$
ENSMUST00000113980	ENSMUSG00000030041	D6Mm5e	11.2	$5.0x10^{-5}$	$7.1x10^{-3}$
ENSMUST00000041703	ENSMUSG00000060962	Dmkn	10.9	$5.0x10^{-5}$	$7.1x10^{-3}$
ENSMUST00000090171	ENSMUSG00000055301	Adh7	10.9	$5.0x10^{-5}$	$7.1x10^{-3}$
ENSMUST00000051176	ENSMUSG00000047420	Fam180a	8.5	$5.0x10^{-5}$	$7.1x10^{-3}$
ENSMUST00000084424	ENSMUSG00000066108	Muc5b	8.3	$5.0x10^{-5}$	$7.1x10^{-3}$
ENSMUST00000090269	ENSMUSG00000068614	Actc1	7.8	$5.0x10^{-5}$	$7.1x10^{-3}$
ENSMUST00000032207	ENSMUSG00000030114	Klrg1	7.5	$5.0x10^{-5}$	$7.1x10^{-3}$
ENSMUST00000113547	ENSMUSG00000023949	Tcte1	7.4	$2.5x10^{-4}$	$2.4x10^{-2}$
ENSMUST00000079400	ENSMUSG00000037686	Aspg	7.4	$5.0x10^{-5}$	$7.1x10^{-3}$
ENSMUST00000020878	ENSMUSG00000020562	Efcab10	7.3	$5.5x10^{-4}$	$4.4x10^{-2}$
ENSMUST00000090568	ENSMUSG00000068748	Ptprz1	6.6	$1.5x10^{-4}$	$1.7x10^{-2}$
ENSMUST00000001384	ENSMUSG00000001349	Cnn1	6.6	$5.0x10^{-5}$	$7.1x10^{-3}$
ENSMUST00000109222	ENSMUSG00000035930	Chst4	6.6	$4.0x10^{-4}$	$3.5x10^{-2}$
**Transcripts Dowregulated in SPC-IGFIR-*Akt2*$^{-/-}$ Tumors**					
Test ID	Gene ID	Gene Name	FoldChange	*p*-val	q-val
ENSMUST00000103420	ENSMUSG00000076614	Ighg1	65.2	$5.0x10^{-5}$	$7.1x10^{-3}$
ENSMUST00000070209	ENSMUSG00000056222	Spock1	31.8	$5.5x10^{-4}$	$4.4x10^{-2}$

(*Continued*)

Transcripts Upregulated in SPC-IGFIR-*Akt2*$^{-/-}$ Tumors					
**Test ID**	**Gene ID**	**Gene Name**	**FoldChange**	***p*-val**	**q-val**
ENSMUST00000032203	ENSMUSG00000030111	A2m	30.7	$1.0x10^{-4}$	$1.2x10^{-2}$
ENSMUST00000173038	ENSMUSG00000092624	Gm3654	22.1	$1.0x10^{-4}$	$1.2x10^{-2}$
ENSMUST00000103397	ENSMUSG00000076596	Igkv3–10	19.8	$5.0x10^{-5}$	$7.1x10^{-3}$
ENSMUST00000103350	ENSMUSG00000076549	Igkv4–68	19.7	$5.0x10^{-5}$	$7.1x10^{-3}$
ENSMUST00000103324	ENSMUSG00000076523	Igkv15–103	16.5	$5.0x10^{-5}$	$7.1x10^{-3}$
ENSMUST00000171139	ENSMUSG00000016283	H2-M2	15.6	$5.0x10^{-5}$	$7.1x10^{-3}$
ENSMUST00000103377	ENSMUSG00000076576	Igkv6–32	15.2	$5.0x10^{-5}$	$7.1x10^{-3}$
ENSMUST00000173446	ENSMUSG00000092312	Zfp419	14.8	$5.0x10^{-5}$	$7.1x10^{-3}$
ENSMUST00000103395	ENSMUSG00000076594	Igkv6–13	14.3	$1.5x10^{-4}$	$1.7x10^{-2}$
ENSMUST00000103520	ENSMUSG00000076711	Ighv8–5	14.1	$5.0x10^{-5}$	$7.1x10^{-3}$
ENSMUST00000103534	ENSMUSG00000076725	Ighv1–63	13.3	$2.0x10^{-4}$	$2.1x10^{-2}$
ENSMUST00000103454	ENSMUSG00000076645	Ighv2–9-1	12.8	$5.0x10^{-5}$	$7.1x10^{-3}$
ENSMUST00000103404	ENSMUSG00000076603	Igkv3–1	12.6	$5.0x10^{-5}$	$7.1x10^{-3}$
ENSMUST00000103528	ENSMUSG00000076719	Ighv8–8	12.3	$5.0x10^{-5}$	$7.1x10^{-3}$
ENSMUST00000103319	ENSMUSG00000076518	Igkv2–112	12.0	$5.0x10^{-5}$	$7.1x10^{-3}$
ENSMUST00000103416	ENSMUSG00000076612	Ighg2c	11.9	$5.0x10^{-5}$	$7.1x10^{-3}$
ENSMUST00000103351	ENSMUSG00000076550	Igkv4–63	11.7	$2.5x10^{-4}$	$2.4x10^{-2}$
ENSMUST00000025636	ENSMUSG00000024730	Ms4a8a	11.2	$5.5x10^{-4}$	$4.4x10^{-2}$

Supplementary Table S3: Diseases and Functions with Z-scores ≥ 2 or ≤-2

Disease or Function	*p*-value	Predicted Activation	z-score	# of molecules
Endothelial cell development	$5.8x10^{-5}$	increased	2.617	21
proliferation of endothelial cells	$7.3x10^{-5}$	increased	2.461	19
metabolism of polysaccharide	$1.4x10^{-6}$	increased	2.399	19
development of endothelial tissue	$3.4x10^{-5}$	increased	2.353	22
synthesis of polysaccharide	$1.5x10^{-6}$	increased	2.272	16
metabolism of carbohydrate	$2.9x10^{-4}$	increased	2.188	30
cell movement of lymphatic system	$3.1x10^{-4}$	increased	2.168	8
colitis	$2.7x10^{-5}$	increased	2.103	18
development of epithelial tissue	$2.1x10^{-4}$	increased	2.102	28
apoptosis of tumor cell lines	$3.8x10^{-4}$	increased	2.064	49
mitogenesis of central nervous system tissue	$2.1x10^{-5}$	increased	2.000	4
colony formation of tumor cell lines	$1.3x10^{-7}$	decreased	–2.464	21
activation of astrocytes	$3.4x10^{-6}$	decreased	–2.377	8
head and neck neoplasia	$2.9x10^{-6}$	decreased	–2.186	84
colony formation of tumor cells	$1.5x10^{-8}$	decreased	–2.091	33
adhesion of immune cells	$2.9x10^{-4}$	decreased	–2.062	20

Supplementary Table S4: Normalized Read Counts for Genes Associated with TAMs

Gene ID	Gene Symbol	SPC-IGFIR tumors	SPC-IGFIR-*Akt2−/−* tumors	Deseq Adjusted pval
ENSMUSG00000000869	*Il4*	0	0	NA
ENSMUSG00000016529	*Il10*	1.2	8	0.65
ENSMUSG00000027776	*Il12a*	3	6.2	0.99
ENSMUSG00000004296	*Il12b*	16	29	0.93
ENSMUSG00000020383	*Il13*	0.5	2.5	0.98
ENSMUSG00000016496	*Pdl1*	592	514	0.99
ENSMUSG00000050737	*Ptges*	5863	3772	0.50
ENSMUSG00000026820	*Ptges2*	2094	1646	0.96
ENSMUSG00000071072	*Ptges3*	3401	4024	0.98
ENSMUSG00000002603	*Tgfb1*	4994	3143	0.47

Supplementary Table S5: Normalized Read Counts for Genes Associated with EMT

Gene ID	Gene Symbol	SPC-IGFIR tumors	SPC-IGFIR-*Akt2−/−* tumors	Deseq Adjusted pval
ENSMUSG00000070473	*Cldn3*	20522	19664	1.00
ENSMUSG00000047501	*Cldn4*	475	1727	0.86
ENSMUSG00000041378	*Cldn5*	1216	3643	0.86
ENSMUSG00000018569	*Cldn7*	7878	6130	0.92
ENSMUSG00000032473	*Cldn18*	30978	37025	0.98
ENSMUSG00000042821	*Snai1*	478	482	1.00
ENSMUSG00000022676	*Snai2*	71	133	0.70
ENSMUSG00000035799	*Twist1*	19	33	0.92
ENSMUSG00000007805	*Twist2*	6	13	0.90
ENSMUSG00000026728	*Vim*	11858	18684	0.72
ENSMUSG00000093585	*Zeb1.1*	1	3	0.98
ENSMUSG00000026872	*Zeb2*	1358	2064	0.61
